# Supplementary material for: From clinics to sewers: leveraging environmental surveillance and whole genome sequencing to inform transmission of ESBL-Escherichia coli in Switzerland
Source: Appl Environ Microbiol. 2026 Apr 6;92(5):e01848-25. doi: 10.1128/aem.01848-25 (PMC13188883; doi:10.1128/aem.01848-25)
Supplement: Supplemental material — Fig. S1 to S8; supplemental table captions. [file aem.01848-25-s0001.docx]

**SUPPLEMENTARY MATERIAL**

**Supplementary figures**

**Fig. S1:** **Regions of Switzerland.**

Map displaying the seven major regions of Switzerland as defined on the Swiss Federal Administration website (<https://www.admin.ch/gov/de/start/dokumentation/medienmitteilungen.msg-id-10585.html>). The regions are Central Switzerland, Eastern Switzerland, Lake Geneva Region, Midland, Northwestern Switzerland, Ticino, and Zurich. The map was generated in R (v4.1.1) and modified in Inkscape (v1.1.1).

**Fig. S2: Distribution of single-nucleotide polymorphism (SNP) differences among replicate sequencing comparisons.** A) Distribution of SNP differences among duplicate pairs, plotting the number of pairwise comparisons (y-axis) against SNP differences (x-axis). These comparisons include 15 ESBL-producing *E. coli* isolates sequenced in duplicate, and two *E. coli* reference strains sequenced multiple times, yielding a total of 96 duplicate pairwise comparisons used to assess sequencing reproducibility. B) SNP differences between a previously sequenced *E. coli* isolate (strain 1) and the same isolate re-sequenced 10 times in the present study. The re-sequenced genomes correspond to the same isolate that contributes to the duplicate comparisons shown in panel A, whereas the original genome was generated in an earlier study using a different DNA extraction, sequencing technology, and bioinformatic processing pipeline. Each comparison represents a pairwise comparison between one re-sequenced genome and the original genome. C) Analogous distribution for a second *E. coli* isolate (strain 2), likewise contributing re-sequenced genomes to the duplicate comparisons in panel A, with each re-sequenced genome (n=9) compared to its original sequence generated independently using a different extraction, sequencing technology, and bioinformatic pipeline. Median SNP differences are indicated by dotted red lines in each panel.

**Fig. S3: Co-occurrence of β-lactamase genes across ESBL-producing *Escherichia coli* isolates stratified by source (clinics, cattle, wastewater, and wildlife).** The heatmap displays individual isolates (rows) and β-lactamase genes (columns). Each tile indicates the presence of a specific gene in a given isolate identified using the Comprehensive Antibiotic Resistance Database (CARD). Genes are grouped and color-coded by gene family: CTX-M (orange), TEM (skyblue), SHV (green), OXA (red), and Other (grey).

**Fig. S4:** **Regional variation in antibiotic resistance genes (ARGs) distribution across clinical, cattle, wastewater, and wildlife sources in Switzerland.** Boxplots show the number of ARGs detected per isolate from clinics (pink), cattle (gold), wildlife (green), and wastewater (blue) across regions of Switzerland. ARGs were identified with Abricate v1.0.1 against CARD (accessed 4 November 2023) and assigned to antibiotic classes using CARD annotations. For the main analysis shown here, we operationally defined clinical relevance as ARGs that are not intrinsic to *E. coli* and not general efflux pumps; genes annotated as general efflux pumps (conferring resistance to at least four antibiotic classes) and genes intrinsic to *E. coli* were excluded from these counts and are summarized separately in Fig. S8. Each panel represents a distinct region, and each boxplot reflects the distribution of ARG counts within a specific source in that region. Only sources with at least three isolates per region are included. *P*-values from the Kruskal-Wallis test are indicated in the bottom left corner of each panel. Significant differences (p-value < 0.05) are marked with *. When *p*-value = NA, the test was not performed due to the presence of only one source category in that region.

**Fig. S5: Distribution of observed and randomized transition counts on the consensus phylogeny.** Transitions between collection sources (clinics, wastewater, wildlife, cattle) were inferred using maximum parsimony ancestral state reconstruction on the consensus maximum likelihood phylogenetic tree of 762 isolates. For each transition type, tip states were randomized 1,000 times (preserving source frequencies) to generate null distributions of transition counts. Each facet shows the distribution of transition counts from the null model (histogram) for a specific transition type. Solid red lines indicate the observed number of transitions; dashed black lines mark the central 95% confidence interval from the null model. Transitions were reconstructed using the *acctran* and *ancestral.pars* functions from the ape package (v5.7.1) in R (v4.1.2).

**Fig. S6:** **Z-score distributions of collection source transitions across 1,000 bootstrap phylogenies for wastewater and clinical isolates (WWC).** Transitions between collection sources (clinics, wastewater) were inferred using maximum parsimony ancestral state reconstruction across 1,000 bootstrap phylogenetic trees, considering only 708 isolates from wastewater and clinical sources. For each tree, tip states were randomized 1,000 times (preserving source frequencies) to generate a null distribution of transition counts. Z-scores represent standardized deviations of observed transitions relative to the null. Each boxplot shows Z-score distributions per transition type, stratified by dataset subset (WWC-only full dataset and the five dominant sequence types: ST131, ST10, ST38, ST69, ST1193, all restricted to WWC isolates). The legend indicates the number of isolates retained in each subset. Red dashed lines mark ±1.96, corresponding to the central 95% confidence interval under the null model. Transitions were reconstructed using the *acctran* and *ancestral.pars* functions from the ape package (v5.7.1) in R (v4.1.2). Positive Z-scores indicate transitions occurring more frequently than expected by chance; negative Z-scores indicate less frequent transitions.

**Fig. S7: Bioinformatic analysis pipeline for ESBL-*E. coli*.** This figure illustrates the bioinformatics workflow used for the analysis of ESBL-producing *Escherichia coli* isolates, implemented through a Snakemake workflow. The pipeline consists of multiple steps: (a) processing 300bp sequencing reads, (b) quality trimming, deduplication, and filtering, followed by (c) de novo genome assembly. (d) Gene detection and annotation were performed, while (e) core genome alignment (99% of isolates) was constructed. (f) Recombinant regions were removed to ensure accurate phylogenetic reconstruction. (g) Pairwise SNP differences were computed, and (h) a maximum-likelihood (ML) phylogenetic tree was generated. (i) Identical sequences from the same sample were removed to prevent redundancy, and (j) transmission events between compartments were quantified.

**Fig. S8: Distribution of antibiotic resistance genes across sources in ESBL-producing *E. coli* isolates.**

This figure shows the proportion of ESBL-*E. coli* isolates carrying genes associated with various antibiotic resistance classes, based on annotations from the Comprehensive Antibiotic Resistance Database (CARD). All resistance genes, excluding β-lactamases, that were detected in at least 5% of isolates from any source are displayed. Each panel represents a different antibiotic class, with colors indicating the sources: clinics (pink), cattle (gold), wildlife (green), and wastewater (blue). Antibiotic resistance classes include aminocoumarins, aminoglycosides, diaminopyrimidines, fluoroquinolones, lincosamides, macrolides, nitroimidazoles, peptides, phenicols, phosphonic acids, rifamycins, sulfonamides, tetracyclines, streptogramins, and disinfectants. When a gene is annotated as conferring resistance to multiple antibiotic classes, it is displayed in all relevant facets. Efflux pumps represent genes conferring resistance to multiple antibiotic classes by actively exporting antibiotics from bacterial cells. Each bar shows the proportion of isolates from a specific source carrying the corresponding gene.

**SUPPLEMENTARY TABLES**

**Table S1:** Summary of the number of isolates collected from various sources, including wastewater, clinics, wildlife, and cattle, along with their respective collection timeframes and locations. The total number of isolates collected from each source category is indicated in parentheses next to the source name.

**Table S2:** Comprehensive summary of ESBL-*E. coli* sequencing outcomes, quality filtering, control types by strain, and resequencing success across batches.

**Table S3:** Summary report of the quality assessment of the genome assemblies conducted using QUAST v5.0.2. The QUAST report, generated as an Excel file, includes various metrics for evaluating assembly quality, such as the number of contigs, total length, N50, L50, and GC content. The report provides a comprehensive comparison of assembly statistics to assess the completeness, accuracy, and overall quality of the genome assemblies.

**Table S4:** Metadata of extended-spectrum β-lactamase (ESBL)-producing *Escherichia coli* isolates collected from wastewater across various regions in Switzerland. The table includes sample identifiers, geographic location (city and region), collection date, detailed source, phylogenetic group, and multi-locus sequence type (ST). These isolates were analyzed to assess genetic diversity, phylogeny, and potential transmission dynamics of ESBL-producing *E. coli* within a One Health framework.

**Table S5:** Distribution of extended-spectrum β-lactamase (ESBL)-producing *Escherichia coli* isolates across nine phylogroups, determined using the EzClermont protocol, stratified by source.

**Table S6:** Distribution of extended-spectrum β-lactamase (ESBL)-producing *Escherichia coli* isolates by sequence type (ST), identified by the analysis of seven housekeeping genes of the multilocus sequence typing (MLST) Achtman scheme using MLST v.2.16.1 (<https://github.com/tseemann/mlst>). The table includes the number and percentage of isolates for each ST.

**Table S7:** Distribution of β-lactamase genes among extended-spectrum β-lactamase (ESBL)-producing *Escherichia coli* isolates from different sources. The table lists detected β-lactamase genes, their presence across sources (clinics, cattle, wastewater, and wildlife), the percentage of isolates carrying each gene, and the corresponding number of isolates. Gene detection was performed by blasting assembled genomes against the Comprehensive Antibiotic Resistance Database (CARD, accessed 4th November 2023) using Abricate v1.0.1.

**Table S8:** Distribution of isolates by number of drug resistance classes. The table shows the number and percentage of isolates resistant to varying numbers of drug classes, based on genes detected from the Comprehensive Antibiotic Resistance Database (CARD, accessed 4th November 2023). Multidrug resistance is abbreviated as “MDR”, and is defined as resistance to three or more classes. Classes considered were: aminoglycosides, β-lactamases, fluoroquinolones, diaminopyrimidines, macrolides, sulfonamides, tetracyclines, phenicols, phosphonic acids, lincosamides, and rifamycins.

**Table S9:** Statistical analysis of differences in antibiotic resistance gene (ARG) number among sources within different regions of Switzerland. The first part of the table presents the results of the Kruskal-Wallis test, indicating the chi-square (*χ^2^*) and p-value for each region: Eastern Switzerland, Midland, Lake Geneva Region, Ticino, and Zurich. The second part of the table shows the results of Dunn’s test with Bonferroni adjustment for pairwise comparisons across all tests, including the Z-score, p-value, and adjusted p-value for each comparison between sources (clinics, cattle, wastewater, and wildlife). Significant comparisons are highlighted in green to indicate regions where sources differ significantly in ARGs number.

**Table S10:** Genetically similar *Escherichia coli* isolate pairs, including extended-spectrum β-lactamase (ESBL)-producing strains, detected within and between compartments across SNP thresholds (0, 1–20, and 21–100 SNPs). The upper section reports total pair counts by comparison type (within vs. between). The middle and lower sections show the percentage and count (n) of genetically similar pairs within and between compartments, respectively. Percentages are calculated relative to the total number of possible isolate pairs within or between the corresponding compartments. Analysis was performed in R (v4.1.1).

**Table S11:** Isolate pairs of extended-spectrum β-lactamase (ESBL)-producing Escherichia coli that were genetically identical (0 SNPs). For each pair, the source compartment, sampling date, and region are shown. The table includes both within-compartment and between-compartment matches; rows shaded in green represent isolate pairs originating from different compartments.

**Table S12:** Significance summary of transition frequencies across 1,000 bootstrap phylogenies for the full dataset and the five most dominant sequence types (ST131, ST10, ST38, ST69, ST1193). The table reports the number of bootstrap trees in which the observed number of transitions between sources fell below, within, or above the central 95% confidence interval of the null distribution generated by 1,000 random permutations. Transitions with counts below the 95% CI (columns "Below 95% CI") or above the 95% CI (columns "Above 95% CI") indicate significant deviation from random expectations at the 0.05 level.

**Table S13:** Significance summary of transition frequencies across 1,000 bootstrap phylogenies based on isolates from wastewater and clinical sources only (WWC). The table reports the number of bootstrap trees in which observed transitions between wastewater and clinics fell below, within, or above the central 95% confidence interval (CI) of the null distribution from 1,000 random permutations. Results are shown for the full WWC phylogeny and for the five most dominant sequence types (ST131, ST10, ST38, ST69, ST1193). Transitions falling below the 95% CI (column "Below 95% CI") indicate significantly fewer transitions than expected under the null model.

**Table S14:** Identical ESBL-*E. coli* isolates (0 SNPs) originating from the same sample and collapsed into a single representative. For each case, the retained isolate is listed alongside the collapsed identical sequences, source compartment, and collection date. This includes, for example, multiple clonal isolates obtained from the same wildlife or livestock fecal sample, or from the same wastewater sample.
